# Factors associated with patient and health care system delay in diagnosis for tuberculosis in the province of Luanda, Angola

**DOI:** 10.1186/1471-2334-13-168

**Published:** 2013-04-08

**Authors:** Luigi Segagni Lusignani, Gianluca Quaglio, Andrea Atzori, Joseph Nsuka, Ross Grainger, Maria Da Conceiçao Palma, Giovanni Putoto, Fabio Manenti

**Affiliations:** 1 Clinical Institute of Hospital Hygiene, Vienna General Hospital, Medical University of Vienna, Währinger Gürtel 18-20, Vienna 1090, Austria; 2 Doctors with Africa CUAMM, Padua, Italy; 3 Department of Innovation, Research and Planning, Azienda ULSS 9, Treviso, Italy; 4 Luanda Tuberculosis and Leprosy Dispensary, Luanda, Angola; 5 National Plan against TB, Luanda, Angola

**Keywords:** Tuberculosis, Delayed diagnosis, System delay, Angola

## Abstract

**Background:**

Tuberculosis (TB) is still a great challenge to public health in sub-Saharan Africa. Most transmissions occur between the onset of coughing and initiation of treatment. Delay in diagnosis is significant to disease prognosis, thus early diagnosis and prompt effective therapy represent the key elements in controlling the disease. The objective of this study was to investigate the factors influencing the patient delay and the health system delay in TB diagnosis in Angola.

**Methods:**

On a cross-sectional study, 385 TB patients who visited 21 DOTS clinics in Luanda were included consecutively. The time from the onset of symptoms to the first consultation of health providers (patients’ delay) and the time from the first consultation to the date of diagnosis (health system’s delay) were analysed. Bivariate and logistics regression were applied to analyse the risk factors of delays.

**Results:**

The median total time elapsed from the onset of symptoms to diagnosis was 45 days (interquartile range [IQR]: 21–97 days). The median patient delay was 30 days (IQR: 14–60 days), and the median health care system delay was 7 days (IQR: 5–15 days). Primary education (AOR = 1.75; CI [95%] 1.06–2.88; p <0.029) and the health centre of the first contact differing from the DOTS centre (AOR = 1.66; CI [95%] 1.01–2.75; p <0.046) were independent risk factors for patient delay >4 weeks. Living in a suburban area (AOR = 2,32; CI [95%] 1.21–4.46; p = 0.011), having a waiting time in the centre >1 hour (AOR = 4.37; CI [95%] 1.72–11.14; p = 0.002) and the health centre of the first contact differening from the DOTS centre (AOR = 5.68; CI [95%] 2.72–11,83; p < 0,00001) were factors influencing the system delay.

**Conclusions:**

The results indicate that the delay is principally due to the time elapsed between the onset of symptoms and the first consultation. More efforts should be placed in ensuring the availability of essential resources and skills in all healthcare facilities other than the DOTS centres, especially those located in suburban areas.

## Background

Angola, a country in south western Africa with a population of 19 million people, was involved in a devastating 27 year-long civil war after gaining its independence in 1975. Despite the end of the civil war and a rapid macroeconomic growth, the country has a high rate of poverty and social inequality [1]. Access to health care is still very limited, and the health status has not improved over time, even following the peace agreement [2]. Angola is divided into 18 provinces. The population of the Luanda province is roughly 5 milllion which represents about 30% of the total population [3].

It is estimated that every year there are 9.4 million new cases of tuberculosis (TB) worldwide and nearly two million deaths from TB [4]. Reservoirs for high levels of TB transmission rest predominantly in those with undiagnosed pulmonary disease [5]. The magnitude of the TB epidemic in sub-Saharan Africa is well recognised. Among other factors, the HIV co-infection has intensified the TB epidemic. Sub-Saharan Africa accounts for approximately 80% of the world’s TB ⁄HIV co-infection cases [4].

TB is one of the common reasons for visits to health facilities in Angola. The country had an estimated incidence rate of TB in 2009 of 298 cases per 100,000 population, with 5.500 deaths (excluding HIV) [4]. The Ministry of Health established the National Tuberculosis Program (NTP) in 1981. Its priority is the establishment of good quality TB diagnostic and treatment services throughout the country. However, its implementation remains weak. Since 1996 the country has adopted the DOTS strategy for treatment. However, in 2007 it only covered 9% of health facilities (138 out of 1,465 facilities) in the country and about 30 percent of the population [2,6,7].

Delays in the diagnosis of TB have been studied in high, middle and low income countries and vary significantly from 63 days in Italy [8] to 88 days in Iran [5] and up to 120 in Burkina Faso [9]. Delay of TB diagnosis may worsen the disease, result in more complications and lead to a higher mortality rate [10]. The contagion parameter suggests that where TB is endemic, each infectious case will result in between 20 and 28 secondary infections [11]. Delay in TB diagnosis may partly explain the high mortality rates among people living with HIV [12]. A recent analysis of TB transmission dynamics and delay has stressed that delays to diagnosis present a major obstacle to the control of a TB epidemic [13]. Factors contributing to delay in diagnosis and treatment are likely to vary depending on the populations in their local settings. Several reviews have analysed factors associated with patient and system delay [14-16]. Thomas (2002) addressed a descriptive analysis of factors related to delay. The study found that associated factors were patient preference for private practitioners (traditional healers, religious healers, pharmacists), a lack of knowledge about TB, stigma and inaccessibility of treatment [14]. The review of Storla *et al.* (2008) emphasised repeated consultations (at either the same level of the system or with the same provider) as a core factor in delay [15]. In a recent review focusing on sub-Saharan Africa, the majority of associations were related to knowledge or enabling factors describing the place of first consultation and travel time and distance from the health facility. Consulting a traditional healer was associated with patient delay [16].

Although operational research has an important role to play in improving the quality and effectiveness of national TB programmes [17], no study has been carried out in Angola. The objective of this operational research is to estimate the diagnostic delays for TB related to the patient or to the health system, analysing possible factors that can influence it. In addition, previous studies have noted that in the assessment of factors associated with system delay, patient sociodemographic characteristics were used to substitute for the lack of system factors [16,18]. Therefore, in the present study several factors more pertinent to the health system (high volume of DOTS centres, density of inhabitants/health care workers, waiting time at the centre) have been analysed.

## Methods

### Study design and study population

We performed a cross-sectional study during a three-month period from April to June 2008. The reference population was composed of patients diagnosed with all forms of TB in DOTS centres (21 in total) with more than 100 registered patients. There were 27 DOTS Centres in the Luanda province. DOTS centres (6 in total) with less than 100 patients registered in 2007 were excluded from the study due to the small size and because they were located in difficult to reach peripheral rural areas. Eight of the 21 DOTS centres were located in the suburban districts of the province (Samba, Cacuaco and Viana), where the population density for each district was less than 900 inhabitants per km^2^ and the nearest health facility was more than 3 km away. Thirteen DOTS centres were settled in the urban districts where the density population was higher and the nearest health facility was less than 2 km away.

According to the NTP, smear-positive pulmonary TB were defined the patients with two or more sputum smears for acid fast bacilli (AFB) or one sputum positive for AFB and radiological abnormalities consistent with active TB; smear-negative PTB were defined the patients with three negative sputum smears for AFB and radiological abnormality consistent with active TB or failure to respond to antibiotics trials. Extrapulmonary TB (EPTB) patients were defined the patients with TB in organs other than the lungs proven by histo-pathology or TB based on strong clinical evidence consistent with active EPTB and the decision by a physician to treat with a full course of anti-TB therapy.

Overall 10.574 TB patients were registered in the 21 DOTS centres in the previous year. The sample size of 385 subjects was determined using the Taro Yamane formula for a finite population [19] and then proportionally stratified to the 21 selected DOTS centres. All patients diagnosed with TB of all forms according to the national TB guidelines [6] and coming to the selected DOTS centres were included consecutively and interviewed just before starting treatment, up to reach the sample size defined for the single centre. Patients who started treatment prior to interview were excluded.

Patient delay was defined as the time from symptom onset to first consultation. Symptom onset referred to the time at which the first symptom (i.e. persistent cough, fever, weakness, weight loss or chest pain) of the illness for which a patient seeking care began. Health system delay was defined from the date of the patient’s first contact with the health care service to the date of diagnosis. Total delay was defined as the sum of patient delay and health system delay. The majority of the quantitative studies measured delay as a dichotomus variable, typically as approximately 1 month or more for patient delay [16,20-22], and 15 days for health system delay [16,21,23,24]. In other studies median value of the observed data was used as a cut-off [24,25]. Following the consultation with experts of the NTP and with treating physicians, we categorised patient delay and health care system delay by using a cut-off point of >30 days and >15 days respectively. In our study the centre of first contact is the first place where the patient seeks health assistance: the TB disease could be suspected in these places, but the TB diagnosis of confirmation was done in the DOTS centres, where the patients received the TB therapy free of charge.

### Survey instrument

A structured questionnaire was developed addressing factors suspected to be involved with health-seeking behaviour based on clinical experience and review of relevant literature (Additional file 1). The questionnaire collected information on the following attributes: i) demographic and socio-economic variables: age, sex, occupation at the time of survey, income before starting treatment, educational level, family size, use of alcohol and tobacco; ii) distance to health facilities, costs of travel to health facility and medical expenditure on treatment of TB, waiting time at the centre (time incurred between arrival at the DOTS centre and receiving therapy); iii) patient knowledge about TB, its cause, gravity and treatment (able to mention bacteria/germ/microbe as a cause of TB, to categorise TB as a transmissible disease, to recognise measures to prevent the transmission, to be able to mention that TB is a treatable disease and the approximate duration of treatment); iv) perception of level and quality of the service received at the DOTS centre (taking into acount the opening time of the centre, the waiting time in the centre, the attitude of the health personnel, do you consider to be; i) definitively satisfied, ii) to some extent satisfied or iii) not satisfied with DOTS centre care received?). The questionnaire was drafted in Portuguese, the official language of Angola. It was pre-tested prior to the start of the study, with 40 patients at the Luanda Lebrosy Dispensary, the province’s biggest DOTS centre. The pre-test allowed the identification of misinterpreted questions. Modifications were then incorporated in the final version. Each interview took place before starting the treatment and lasted approximately 20 minutes. Three trained interviewers carried out the fieldwork under the supervision of a physician. A written consent was secured from every eligible tuberculosis patient before inclusion into the study. Informed consent and responses for children under 18 years of age were obtained from the closest accompanying relative. The study was approved by the ethics committees of the Health direction of Luanda province.

### Data analysis

Data were analysed using SPSS for windows version 17. Beyond descriptive statistics, associations between the dependent variables (patient delay and health system delay) and the independent variables were analysed by calculating the ORs test and respective 95% confidence intervals (CI). Responses to questions to assess TB knowledge were analysed by calculating their mean and interquartile scores. Using the mean score as a cut-off, the responses were categorised into good knowledge (above or equal the mean) and poor knowledge (below the mean) [26].

Independent variables that showed marginal associations (p < 0.20) in the bivariate analysis were used in a logistic regression analysis in order to identify independent predictors of both patient delay > 30 days and health system delay > 15 days. The association of predictor variables with the dependent variables was assessed by using 95% Cl and adjusted odds ratio (AOR). A p-value of <0.05 was regarded as statistically significant.

## Results

### Socio-economic characteristics of study participants

During the study 385 individuals were enrolled. There were 229 males and 156 females, with a median age of 29 years (IQR 22–39) (Table 1). Pulmonary TB patients were 377, among them 262 were smear positive and 123 smear negative. There were 8 EPTB patients, one of whom was smear positive and seven were smear negative. The median income per month was US$ 100 (IQR 0–200). The level of education and employment is significantly higher among males than females (p < 0.01), as is income (p < 0.05). The median number of household members was six (IQR 4–8). Out of the total, 277 (72%) lived in urban areas and 108 (28%) in suburban areas. 262 (68%) patients had acid-fast bacilli in their sputum (Table 2). Median (IQR) transport time to reach the TB centre was 30 minutes (30–60) and the median cost of transport was US$ 0.67 (0–1,33). Median waiting time at the centre was 15 minutes (IQR 10–30).

**Table 1:** Socio-demographic characteristics of participants (=385 subjects)

**Characteristics**	**Patients (n.)**	**Patients (%)**
***Gender***		
Male	229	59
Female	156	41
M/F ratio	1,467	
***Age (years)***		
*Median (interquartile range)*	*29*	*(22–39)*
1–15	38	10
15–30	187	49
31–45	116	30
>45	44	11
***Education***		
Illiterate	190	49
Primary	121	31
Secondary	74	19
***Income (per month in US $)***		
*Median (interquartile range)*	*100*	*(0–200)*
None	155	40
1–200	138	36
201–400	69	18
> 400	23	6
***Occupation***		
Employed	89	23
Unemployed	296	77
***N. household members***		
*Median (interquartile range)*	*6*	*(4–8)*
1–4	122	32
5–8	197	51
≥ 9	66	17
***Residence***		
Urban	277	72
Suburban	108	28
***Alcohol use***		
Yes	201	52
No	184	48
***Tobacco use***		
Yes	109	28
No	276	72
***Transport method***		
Vehicle	219	57
Walking	166	43
***Time taken to reach the TB centre***		
*Median (interquartile range)*	*30*	*(30–60)*
≤1/2 hour	208	54
>1/2– ≤ 1 hour	119	31
>1 hour	58	15
***Transport cost (in US $)***		
*Median (interquartile range)*	*0,67*	*(0–1,33)*
None	171	44
≤ 1	74	19
1–1,9	81	21
≥2	59	15

**Table 2:** Diagnostic and clinic factors of participants

**Factors**	**Patients (n.)**	**Patients (%)**
***Sputum microscopy***		
Positive	262	68
Negative	123	32
***Basic knowledge of TB***		
Yes	236	61
No	149	39
***Expenses for diagnosis/treatment***		
Yes	227	59
No	158	41
***Centre of first contact***		
DOTS Centre	126	33
Hospital	122	32
Health care provider	72	19
Private health worker	50	13
Self-medication	9	2
Traditional healer	6	2
***High volume DOTS centres***		
< 600 patients	164	43
≥ 600 patients	261	57
***Density inhabitants/HCW (physicians and nurses)***		
*<1400*	197	51
*≥1400*	188	49
***Waiting time at in the centre for being attended***		
*Median (interquartile range)*	*15 minutes*	*(10–30)*
≤ 1/2 hour	296	77
> 1/2– ≤ 1 hour	57	15
> 1 hour	32	8
***Satisfaction with DOTS Centre care received***		
Definitely satisfied	358	93
To some extent satisfied	25	6
Not satisfied	2	1
No answer	0	0
***Food in the DOTS centre***		
Yes	29	8
No	356	92
***Education in the DOTS centre***		
Yes	298	77
No	87	23

### Patients’ delay

**Figure 1:**
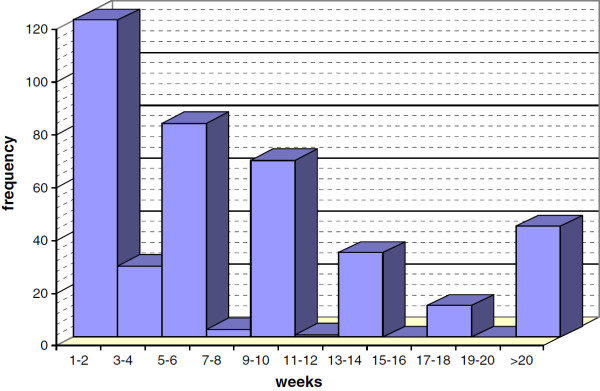
**Patient delay in weeks.** This figure shows the time intercourring between the onset of symptoms and help seeking: among 385 surveyed patients, 147 reported their symptoms to a health provider within 4 weeks and 238 after 4 weeks.

Patient delay had a median value of 30 days (IQR 14–60 days) and a mean value of 71.37 days (SD 133.94). The distribution of the reported duration of symptoms prior to seeking help is shown in Figure 1. Factors potentially associated with prolonged patient delay were analysed using bivariate analysis for delays of over four weeks, as shown in Table 3. Factors associated with delay included primary education, volume of DOTS centres ≥ 600 patients per year, no food in the DOTS centre and health centre of the first contact if this was different from the DOTS centre. In the logistic regression analysis, primary education (AOR = 1.75; CI (95%) 1.06–2.88; p <0.029) and health centre of the first contact if this was different from the DOTS centre (AOR = 1.66; CI (95%) 1.01–2.75; p <0.046) remained independent risk factors for patient delay >4 weeks.

**Table 3:** Factors associated with patient and health system delay: bivariate analysis

**Variable**	**Patient delay**			**System delay**		
	**OR**	**CI (95%)**	**p**	**OR**	**CI (95%)**	**p**
***Sex***						
Males	1			1		
Females	1,22	0,80–1,84	0,3543	0,96	0,58–1,58	0,8734
***Age***						
1–15	1			1		
16–30	1,25	0,61–2,58	0,5374	0,93	0,39–2,18	0,8588
31–45	0,97	0,45–2,08	0,9437	1,14	0,47–2,77	0,7765
>45	1,71	0,71–4,15	0,2312	1,25	0,44–3,52	0,6727
***Education***						
None	1			1		
Primary	1,97	1,24–3,14	0,0039^*^	0,88	0,51–1,53	0,6527
Secondary/Univ.	1,21	0,70–2,11	0,4984	0,62	0,31–1,26	0,1853
***Income***						
None	1			1		
1–200	0,99	0,62–1,58	0,9575	1,21	0,68–2,14	0,5186
201–400	1,54	0,87–2,73	0,139	1,65	0,85–3,21	0,1372
> 400	1,22	0,50–2,95	0,6622	1,21	0,41–3,52	0,7302
***Occupation***						
Employed	1			1		
Unemployed	0,71	0,44–1,15	0,1604	0,73	0,42–1,27	0,2623
***N.household members***						
1–4	1			1		
5–8	0,86	0,55–1,37	0,5316	0,88	0,50–1,54	0,6612
≥ 9	0,99	0,54–1,82	0,979	1,49	0,75–2,97	0,2515
***Residence***						
Urban	1			1		
Suburban	1	0,63–1,57	0,9923	1,87	1,12–3,12	0,0162^*^
***Alcohol use***						
Yes	1			1		
No	0,88	0,58–1,32	0,5288	1,09	0,67–1,76	0,7409
***Tobacco use***						
Yes	1			1		
No	0,87	0,56–1,37	0,5571	1,04	0,4–1,79	0,8909
**Transport method**						
Vehicle	1			1		
Walking	0,85	0,56–1,28	0,4393	0,89	0,55–1,46	0,6547
***Time taken to reach the TB centre***						
≤1/2 hour	1			1		
>1/2– ≤ 1 hour	1,16	0,73–1,83	0,5274	1,46	0,85–2,51	0,1684
>1 hour	1,39	0,78–2,51	0,2665	1,38	0,69–2,76	0,3637
***Transport cost in US $***						
None	1			1		
≤ 1	1,35	0,78–2,35	0,2825	1,03	0,53–2,01	0,9203
1–1,9	1,15	0,67–1,97	0,6086	1,00	0,52–1,91	0,9906
≥2	1,02	0,55–1,87	0,9581	1,17	0,58–2,36	0,6674
***BK***						
Negative	1			1		
Positive	1,06	0,68–1,64	0,7967	1,04	0,61–1,75	0,8907
***Basic knowledge of TB***						
Yes	1			1		
No	0,88	0,58–1,34	0,5566	1,06	0,64–1,74	0,8232
***Expenses for diagnosis/treatment***						
No		1		1		
Yes	1,22	0,80–1,85	0,3502	0,94	0,58–1,54	0,8132
***High volume DOTS centres***						
<600 patients	1			1		
≥600 patients	0,59	0,39–0,89	0,0125^*^	0,75	0,46–1,22	0,2468
***Density inhabitants/HCW (physicians and nurses)***						
<1400	1			1		
≥1400	0,89	0,59–1,34	0,5803	1,16	0,72–1,89	0,5403
***Waiting time at in the centre for being attended***						
≤1/2 hour	1			1		
>1/2– ≤ 1 hour	0,62	0,34–1,11	0,107	1,29	0,66–2,52	0,4478
>1 hour	0,59	0,27–1,29	0,1816	2,49	1,15–5,54	0,0172*
***Food in the DOTS centre***						
Yes	1			1		
No	2,82	1,12–7,10	0,0221*	0,58	0,25–1,33	0,1969
***Education in the DOTS centre***						
Yes	1			1		
No	0,66	0,40–1,10	0,1082	1,21	0,69–2,13	0,506
***Centre of first contact***						
DOTS centre	1			1		
others	1,97	1,25–3,09	0,0031^*^	4,03	2,05–7,91	<0,00001^*^

* : p value < 0,05.

### Health systems’ delay

**Figure 2:**
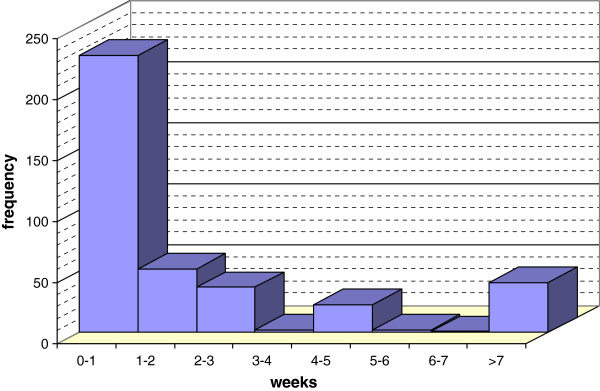
**Health system delay in weeks.** This figure shows the time elapsed between the first contact with the health provider and the diagnosis: among 385 surveyed patients, 279 had a health system delay less than 2 weeks and 106 more than 2 weeks.

The median health system delay was seven days (IQR 5–15) and its mean value was 28.66 days (SD 82.38). The distribution of the reported duration of system delay is shown in Figure 2. Of the factors investigated, these were the three independent predictors of health service delay >2 weeks, after adjusting for other variables (Table 3) namely: living in a suburban area (AOR = 2,32; CI (95%) 1.21–4.46; p = 0.011) waiting time in the centre >1 hour (AOR = 4.37; CI (95%) 1.72–11.14; p = 0.002) and the centre of the first contact differening from the DOTS centre (AOR = 5.68; CI (95%) 2.72–11,83; p < 0,00001). In the logistic regression analysis, the centre of the first contact differening from the DOTS centre showed the following results: hospital AOR = 17.66; CI (95%) 1.1–284,42 (p = 0.043); health care provider AOR = 35.57; CI (95%) 2.37–533.1 (p = 0.01); private health care worker AOR = 1.90; CI (95%) 1.06–3.43 (p = 0.032); self-medication AOR = 1.98; CI (95%) 1.00–3.90 (p = 0.05).

We analysed if the system delay was influenced by the patient delay: the chi-square test was not significant (p = 0.12).

### Total delay

Among the 385 subjects, the median total delay was 45 days (IQR 21–97) and the mean total delay was 100.43 days (SD 100.43). For 61 (15%) subjects, the total delay was ≥ 20 weeks. The chi-square test between subjects with a total delay < 20 weeks and subjects with a total delay ≥ 20 weeks resulted not significant for the variables considered.

## Discussion

The key elements in any TB control programme are the early dagnosis and prompt institution of effective treatment. Focus on patient and health system (provider and health facility) factors associated with delay is an important starting point to identify how to improve TB control. We assessed delay and associated factors in the diagnosis of TB in Luanda, Angola.

### Patients’ delay

In our setting the patient delay is 30 days and four times higher than the system delay. In a recent review Sreeramareddy *et al.*[18]*,* found that among the low and middle income countries patient delay varied from 4.9 days in Gambia [27] to 162 days in Tanzania [20]. The average patient delay in low and middle income countries was 31.7 days. It is likely that most studies underestimate the duration of illness/disease before diagnosis, since patients often do not clearly recognise symptoms at the onset of the disease. This has been demonstrated in several recent TB prevalence surveys, in which less than half of the cases fulfil the criteria of being a “TB suspect” and many have only vague symptoms [28]. One can debate if such delay is a patient delay (if a person does not feel sufficiently ill, why take action?) or a delay in diagnosis caused by the nature of the disease. In the current study, only one socio-demographic characteristic appeared to correlate with patients’ delay, namely primary education. Primary educated patients experienced much longer diagnostic delays than those fewer years of education. However, this data is contradicted in patients with secondary education. Perhaps in a proportion of the population primary education rates do not translate into greater awareness of TB. Although higher levels of education can be considered an indicator of TB knowledge, recent evidence suggests that TB treatment initiation is more influenced by perceptions than by knowledge [29]. Lay perceptions may explain why people continue and others may stop taking treatment and this regardless of their educational level [29,30]. The acknowledgement of social, economic and geographical context is for sure necessary to understand the impact of traditional beliefs and perceptions of illness [31,32]. Several studies for example show that people have traditional beliefs about TB [32-34]. Khan *et al.,* (2000) found no difference in treatment outcome related to educational levels in Pakistan [35]. In Ethiopia, high default rates were related to a lack of family support, inadequate knowledge and medication side effects [36]. In addition, sex and gender differences have been reported [33,37].

Since 2007 the Angolan NTP has been implementing a plan with the main objective to increase TB awareness through advocacy, communication and social mobilisation activities [7]. This must be strengthened in the future, improving both attitudes and TB perceptions.

Another factor associated with prolonged patient delay was having a first point of contact that was different from the DOTS centre. One could hypothesize that those who go to a centre different than the DOTS centres are less educated people or people with less knowledge of the disease: the chi-square test did not confirm this assumption (p = 0,3448 for knowledge and p = 0,08 for education respectively). With the onset of symptoms, patients initially practised self medication, visited traditional healers, or resorted to non-prescribed medications from pharmacies. Meanwhile, they would also seek care at a health care provider. These different actions varied across countries [5].

In the present study, the use of private sector, including traditional healers, remains relatively minor as a point of first contact (15%), although its progression should be monitored. The use of traditional healers as the preferred source of care was also low, only 2%. However, this percentage may be reasonable considering the urban-suburban focus of the study. Previous works conducted in sub-Saharan Africa had suggested a higher preference for traditional healers, particularly for people who live in rural areas [20,21,38]. We found that only 2% reported self medication as first action, a low percentage compared to other studies. The fact that hospitals were the first point of care sought by approximately one third of patients reflects the poor quality of the primary health care system in Luanda.

In contrast 33% said they went directly to a DOTS centre, which is high compared to other studies [5,27]. A number of patients have probably been elsewhere before coming to the DOTS centre, but they are not reporting it. This may be due to patients’ reluctance to express their preference freely in a DOTS setting, despite interviewers’ efforts to investigate this aspect in an open manner [39]. Collaboration with hospitals, health care providers, and private health workers is therefore important to reduce enrolment delays in DOTS programmes in Luanda. The free services of the DOTS programmes should probably be made known more widely across the community.

### Health system’s delay

In the current study the system delay was very short, suggesting that referrals and diagnosis were swift whenever a person with TB interacted with the health system. However, as we have pointed out, there might be problems validating information about the place of first contact with the health system, especially when it was made outside the public sector. Despite this positive result, rapid diagnostic tests have to be used in the care process. One such test, the newly developed Xpert MTB/RIF, needs implementation studies in low income countries [40]. Similarly, the effectiveness of existing tools needs to be assessed [41].

Living in suburban area was a factor associated with prolonged system delay (median system delay in sub urban area 14 days (IQR 7–30); median system delay living in urban area 7 days (IQR 5–14)). A similar conclusion emerged from other studies [23]. Despite the fact that suburban inhabitants are likely to have poorer access to health care [21,42-44], interestingly there was no increase in the patient delay in this study compared to urban dwellers. Although the number of positive smear tests was not significantly lower in suburban areas (87/123 in suburban area, vs 190/262 in the urban area, p = 0.71) this suggests that the suburban health centres are poor at diagnostic TB. Management and provision of resources for diagnosis of TB in Luanda province has probably been over-centralised, with the result that suburban dwellers have been disadvantaged.

Although the median waiting time in the health centre was acceptable (15 minutes), 8% of patients declared that they had to wait more than one hour. To the best of our knowledge, this is the first time that waiting times at the centre resulted in system delay. This is probably related to the fact that few studies have analysed the health system factors responsible for delay in diagnosis, frequently using data related to sociodemographic characteristics of patients. With the increase of HIV treatment in sub-Saharan Africa it will be important to facilitate an integrated approach to the management of co-infected persons, creating a “one stop” service in order to facilitate dually infected patients to be treated for both conditions simultaneously. By providing a “one stop” service patients attend one rather than two consultations, which reduces waiting time.

If the centre of first contact differed from the DOTS centre, this was a factor associated both with prolonged patient and system delay. The median patient delay for DOTS centre is 21 days, vs. the 30 days of the non DOTS centre as a whole. Among non-DOTS centres, the median patient delay increased to 33.5 days for traditional healers and up to 37.5 days for the hospital. It seems that the DOTS centres, in comparison to the others centres, are capable of facilitating the first contact with the patient. DOTS centres seem to perform better also in relation to the time needed for diagnosis. The median system delay for the DOTS centres is 5 days which increased to 7 days for non DOTS centres as a whole. The median system delay for hospitals and health care providers is in line with these figures (7 and 8 days respectively), but system delay increased to 14 days for private care providers, 40.5 days for traditional healers and 90 days in cases of self medication.

A final comment on system delay is that it is probably needed to be more inclusive in the definition of a “TB suspect”. Normally, national TB programmes recommend that people with chronic cough (>2–3 weeks) should be tested for TB, but that is missing with many diseases. This is also the norm in Angola.

### Total delay

In the present study the total time elapsed between symptom onset and diagnosis of TB (45 days) was similar or lower than the delay reported in the majority of other studies carried out in sub-Saharan Africa [9,21-24,27,38,39,45-48]. In these studies total delays ranged from 6 to 16 weeks.

The present study has certain strengths: it involved 21 of the 27 DOTS centres in Luanda province; the questionnaire had been previously tested in a pilot study, which allowed us to improve the instrument. Finally, it is the first on this issue carried out in Angola. However, our study has some limitations. The questionnaire used did not quantitatively address several items such as smoking and alcohol, and did not attempt to explain why the diagnosis was delayed. For measuring the length of patient delay, we depended on the reply of the patient. Consequently, some patients may not remember the exact onset of symptoms. In addition we do not have information on the HIV serostatus of the patients. Although no studies in sub-Saharan Africa have reported a link between HIV and a delay in the diagnosis of TB, this is a condition that needs additional research in the future [22,46]. Another limitation of the current study is that we are not able to provide data on laboratory turn-around time. So it is not possible to say if the lab plays an important role in the in health system delay. Finally, although for the health centres we have tried to analyse several factors focused more on system delay other than the socio-demographic characteristics of patients, part of the factors analysed are related to socio-demographic and clinical characteristics of patients. As already observed by Finnie *et al.*[16], these would be considered predisposing factors for system delay in the sense that they may be related to the aspect of system delay in which patient return for diagnosis is required; they also may indicate health care workers’ perceptions of patients that may cause them to be less likely to test and diagnose TB [16]. Anyhow, in future research factors more pertinent to the health system have to be studied in a better way [16,18].

## Conclusions

The diagnosis of TB is delayed in the province of Angola mainly because of the time elapsed between the onset of symptoms and the first consultation, especially among people with primary education and with a first point of contact that is differens from the DOTS centre. Future qualitative studies should be conducted in order to clarify the reasons for the delay in diagnosis. We recommend that the NTP strengthen the educational campaigns designed to provide information regarding the symptoms of TB, maybe focusing on improving attitudes and perceptions. Moreover, collaboration with hospitals, health care providers and private health workers is important to reduce enrolment delays in DOTS programmes in Luanda. Waiting times in the centre >1 h, sub urban health centres and the centre of the first contact differeing from the DOTS were responsible for the long health service delays. It indicates the need for regular supervision and ensuring the availability of essential resources and skills, particularly at sub urban health facilities and health centres differing form the DOTS centres in order to ascertain their proper functionality and level of quality of available services. In the future comparable operational research could be useful for monitoring improvement in the quality of the TB control program in Angola.

## Competing interest

The authors declare that they have no competing interests.

## Authors’ contributions

LSL, JN, MCP, and AA designed the study. FM, LSL, GP, and JN conceived of the study, and participated in its design and coordination. LSL and GLQ conducted the statistical analyses. GLQ, LSL, GP and FM drafted the manuscript and incorporated all suggestions. All authors made significant contributions to the conception and design of the analyses, interpretation of the data, and drafting of the manuscript, and all authors approved the final manuscript.

## Pre-publication history

The pre-publication history for this paper can be accessed here:

http://www.biomedcentral.com/1471-2334/13/168/prepub

## Supplementary Material

Additional file 1 The questionnaire. Click here for file
