# Supplementary material for: Factors associated with patient and health care system delay in diagnosis for tuberculosis in the province of Luanda, Angola
Source: BMC Infect Dis. 2013 Apr 8;13:168. doi: 10.1186/1471-2334-13-168 (PMC3637285; doi:10.1186/1471-2334-13-168)
Supplement: Additional file 1 — The questionnaire. [file 1471-2334-13-168-S1.pdf]

QUESTIONÁRIO PARA DOENTES DE TB EM TRATAMENTO

N° questionário: ....

Centro Dots: .... Data / /

N° Proceso: .... N° entrevistador: ....

**1.** Sexo	☐ Homem	☐ Mulher
**2.** Idade	.... anos	
**3.** Categoria Doente	☐ Categoria 1 ☐ Categoria 3<br>☐ Categoria 2 ☐ Categoria Criança *(<16 anos)*	
**4.** BK (baciloscopia)	☐ Positiva	☐ Negativa ou Não realizada
**5.** Classificação Doença	☐ Pulmonar	☐ Extra Pulmonar
**6.** Inicio tratamento	........../........../..........	
**7.** Profissão (Condição laboral)	☐ Empregado	☐ Não empregado
**8.** Qual è o seu salário (*ou* era se deixou por causa da doença)?	.... Kwanza por mês *ou*<br>............................. Kwanza por dia *ou*<br>............................. Kwanza por semana *ou*<br>............................. Kwanza por ano	
**9.** Qual è a sua escolaridade?	☐ Nenhuma ou deixou antes do diploma<br>☐ Diploma de ensino primário<br>☐ Diploma de ensino médio (secundário)<br>☐ Diploma universitário (superior)<br>☐ Outro (..)	
**10.** Quantas pessoas vivem na sua casa (*incluindo você*)?	n°...............	

**11.**Fuma?	☐ sim ☐ nunca fumou<br>☐ deixou desde o inicio do tratamento
**12.**Bebe álcool?	☐ sim ☐ nunca bebeu<br>☐ deixou desde o inicio do tratamento
**13.**Quanto tempo demora para chegar ao Centro?	☐ .... minutos ☐ .... horas
**14.**Quanto tempo demora para regressar a casa ou trabalho?	☐ .... minutos ☐ .... horas
**15.**Quanto tempo espera para ser atendido no Centro?	☐ .... minutos ☐ .... horas
**16.**Que meio de transporte utiliza para chegar ao Centro?	☐ Carro privado ☐ Autocarro<br>☐ Candongueiro-taxi ☐ Motorizada<br>☐ A pe ☐ Outros (...........................)
**17.**Quanto custa o transporte para chegar ao Centro? *(só ida)*	.... Kwanza
**18.**Desde o inicio do tratamento, teve que pagar para outros exames (*Raios X, análises do laboratório, do sangue, etc.*)	☐ sim ☐ não
**19.**Se sim, quanto pagou no total até agora?	.... Kwanza
**20.**Desde o inicio do tratamento, já aconteceu que teve comprar medicamentos para TB?	☐ sim ☐ não
**21.** Se sim, quanto pagou no total até agora?	.... Kwanza
**22.**No centro, recebeu educação sanitária sobre a TB?	☐ sim ☐ não
**23.**Antes de vir ao Centro DOT foi à procura de outros tratamentos?	☐ sim ☐ não
**24.**Se sim onde?	☐ auto-medicação ☐ curandeiro (medicina tradicional)

	☐ medico privado ☐ enfermeiro particular<br>☐ hospital ☐ centro de saúde<br><br>☐ outros<br>(…………………………………………………………………)
**25.** Quanto tempo ficou em casa antes de ter ido a consulta (*a primeira*), ficando com sintomas da doença *(tosse,febre,dor torácico,falta de apetite,perca de peso)*?	☐ ……. dias ☐ ……. semanas ☐ ……. meses
**26.** Quanto tempo passou entre este primeira consulta e o inicio do tratamento nesse Centro DOT?	☐ ……. dias ☐ ……. semanas ☐ ……. meses
**27*(a)*** Qual é a causa da tuberculose?***(R: Bacilo ou Bactéria ou microbo)***	☐ correcta ☐ não correcta
**27*(b)*** Como se evita a transmissão da doença às outras pessoas?<br>***(R: pôr as mãos á boca quando tosse ou espirra; cuspir em contendores; ventilar os ambientes fechados onde esteve)***	☐ sabe ☐ não sabe
**27*(c)*** Por quanto tempo deve tomar o tratamento antituberculose?<br>***R: Categorias I,II,III: 8 meses***<br>***Categoria Criança: 6 meses***	☐ sabe ☐ não sabe
**28.** Como julga a qualidade do serviço recebido no Centro?	☐ Boa ☐ Aceitável<br><br>☐ Não boa ☐ Não responde
**29.** O Centro entrega-lhe incentivos alimentares?	☐ sim ☐ não
**30.** Qual è o seu maior impedimento para chegar cada dia ao Centro?	☐ Tempo para a viagem<br>☐ Custo da viagem<br>☐ Indisponibilidade de acompanhantes<br>☐ Horário de abertura do Centro<br>☐ Demora prolongada no Centro<br>☐ Atitude da equipa sanitária do Centro<br>☐ Efeitos secundários dos medicamentos<br>☐ Estigma (Vergonha) da doença<br>☐ Problemas de saúde<br>☐ Outros<br>(……………………………………………………………)<br>☐ Não responde

**31.** *(Só se NÃO empregado)* Teve que deixar o trabalho por causa da tuberculose?	☐ sim	☐ não
**32.** *(Só se empregado)* Você tem que faltar ou chegar tarde ao trabalho para vir ao Centro?	☐ sim	☐ não
**33.** *(Só se empregado)* Precisa de autorização do chefe do trabalho para vir ao Centro?	☐ sim	☐ não
**34.** *(Só se empregado)* Se sim, recebe a permissão facilmente?	☐ sim	☐ não
**35.** Habitualmente chega ao Centro acompanhado por alguém?	☐ sim	☐ não
